# Oncogenic KRAS activates an embryonic stem cell-like program in human colon cancer initiation

**DOI:** 10.18632/oncotarget.6818

**Published:** 2016-01-04

**Authors:** Anne-France Le Rolle, Thang K. Chiu, Zhaoshi Zeng, Jinru Shia, Martin R. Weiser, Philip B. Paty, Vi K. Chiu

**Affiliations:** ^1^ Division of Hematology/Oncology, Department of Medicine, University of California, Irvine, CA, USA; ^2^ Chao Family Comprehensive Cancer Center, University of California, Irvine, CA, USA; ^3^ Department of Biochemistry and Molecular Biology, Louisiana State University Health Sciences Center, New Orleans, LA, USA; ^4^ Department of Surgery, Memorial Sloan-Kettering Cancer Center, New York, NY, USA; ^5^ Department of Pathology, Memorial Sloan-Kettering Cancer Center, New York, NY, USA

**Keywords:** KRAS, embryonic stem cell-like program, colorectal cancer, cancer plasticity, EMT

## Abstract

Colorectal cancer is the third most frequently diagnosed cancer worldwide. Prevention of colorectal cancer initiation represents the most effective overall strategy to reduce its associated morbidity and mortality. Activating KRAS mutation (*KRAS^mut^*) is the most prevalent oncogenic driver in colorectal cancer development, and *KRAS^mut^* inhibition represents an unmet clinical need. We apply a systems-level approach to study the impact of *KRAS^mut^* on stem cell signaling during human colon cancer initiation by performing gene set enrichment analysis on gene expression from human colon tissues. We find that *KRAS^mut^* imposes the embryonic stem cell-like program during human colon cancer initiation from colon adenoma to stage I carcinoma. Expression of miR145, an embryonic SC program inhibitor, promotes cell lineage differentiation marker expression in *KRAS^mut^* colon cancer cells and significantly suppresses their tumorigenicity. Our data support an *in vivo* plasticity model of human colon cancer initiation that merges the intrinsic stem cell properties of aberrant colon stem cells with the embryonic stem cell-like program induced by *KRAS^mut^* to optimize malignant transformation. Inhibition of the embryonic SC-like program in *KRAS^mut^* colon cancer cells reveals a novel therapeutic strategy to programmatically inhibit *KRAS^mut^* tumors and prevent colon cancer.

## INTRODUCTION

Colorectal cancer is the third most frequently diagnosed cancer worldwide and leads to death in one-third of afflicted adults. Colorectal carcinomas arise from adenomas, which are found in >25% of adults undergoing initial screening colonoscopies at age 50 [1]. Since only <5% of colorectal adenomas will develop into carcinomas, the most effective strategy to decrease colorectal cancer mortality is to understand and prevent the malignant transformation process. While it is known that *APC* or *CTNNB1* mutation activates aberrant WNT signaling to give rise to colon adenomas, the programs that promote their transformation to carcinomas are not well characterized. Additional driver mutations such as oncogenic KRAS occur in the context of enlarging colon adenomas to enhance colon cancer initiation [2]. The current model suggests that oncogenic KRAS promotes the malignant transformation of colon adenoma to carcinoma through further hyperactivation of WNT signaling [3, 4].

Activating mutations of KRAS (*KRAS^mut^*) are among the most prevalent oncogenic mutations in human cancers and occur in approximately half of human colorectal cancer [5]. *KRAS^mut^* has reduced GTPase activity and accumulates in the active GTP-bound state, which results in sustained signaling of downstream pathways regulating cell proliferation and survival. Notably, *KRAS^mut^* is a predictive biomarker of resistance to EGFR-targeted monoclonal antibody therapy and leads to a dangerous selection process for tumor recurrence and metastasis [6]. Despite great advances in understanding RAS signaling and regulation, prior attempts to inhibit tumor growth by direct inhibition of oncogenic RAS has remained clinically unproven [7]. The individual or combined targeting of downstream effectors of RAS, such as MEK and AKT, cannot systematically bypass the signaling redundancy inherent in colorectal cancer growth [8, 9]. The importance and difficulty of this challenge has been recognized by the US National Cancer Institute, which has launched the RAS initiative as a concerted effort to develop novel strategies to modulate oncogenic KRAS activities.

Crypt base intestinal stem cells (SC) in the normal colon epithelia are regulated by WNT signaling, express the WNT target gene leucine-rich-repeat containing G-protein-coupled receptor 5 (*LGR5*), and differentiate as they migrate up the crypt axis to give rise to all colon epithelial cells [10, 11]. The finding that murine intestinal SC with aberrant WNT signaling may give rise to colon adenomas highlights the importance of intestinal SC signaling in colon adenoma pathogenesis [12]. Human colon adenoma development also depends on the activation of aberrant WNT signaling [13], which is presumed to hyperactivate the intestinal SC program that promotes further malignant transformation to colon carcinoma based on the current model [14]. However, the contribution of other SC programs to colon cancer initiation is unknown. In non-colorectal epithelial cancers, activation of the embryonic SC-like program was associated with poor differentiation when compared to well differentiation [15]. Here we examine the impact of distinct SC programs in colon cancer initiation, which presents predominantly with moderate differentiation (>70%). Since analysis of differential gene expression programs by gene set enrichment analysis (GSEA) has proven crucial in identifying biologically meaningful patterns at the systems-level, we performed GSEA of human colon tissues [16]. We find that *KRAS^mut^* accelerates colon cancer initiation from a pre-malignant adenoma into a malignant stage I carcinoma by imposing the embryonic SC-like program. Inhibition of *KRAS^mut^* colon tumors with miR145, an embryonic SC inhibitor, suppresses their malignant growth. These data elucidate the embryonic SC-like program as a novel and targetable system-level mechanism for *KRAS^mut^* mediated tumorigenicity.

## RESULTS

### Activation of the embryonic SC-like signature during transition from adenoma to carcinoma

**Figure 1:**
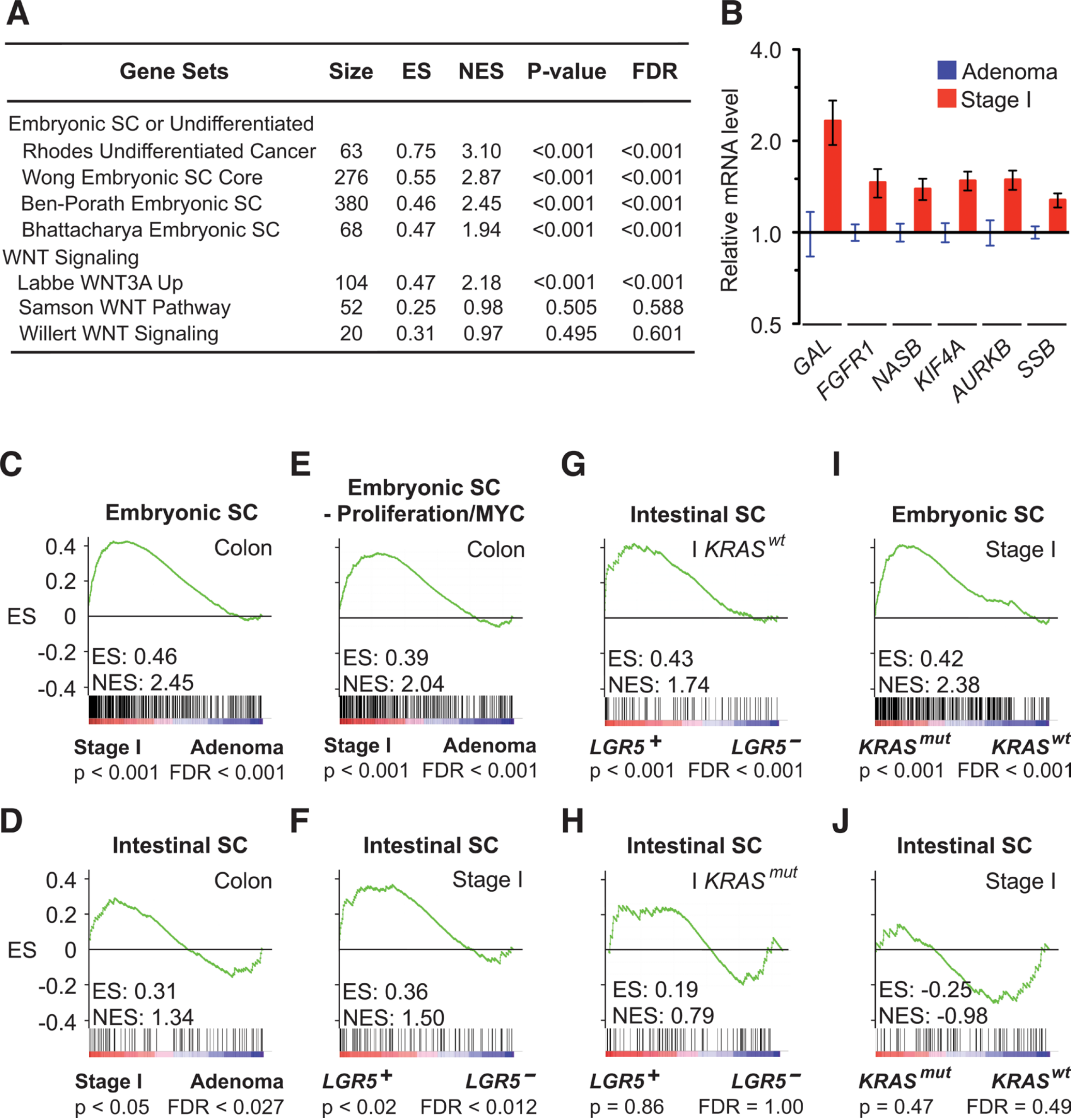
*KRAS^mut^* and LGR5 induce distinct embryonic SC-like and intestinal SC programs **A**. GSEA comparing human stage I colon carcinomas to colon adenomas of selected stem cell signatures from MSigDB C2: chemical and Genetic Perturbations gene sets (Broad Institute). **B**. Expression of embryonic SC genes in human stage I colon carcinomas relative to colon adenomas. **C**. and **D**. GSEA on human stage I colon carcinomas versus adenomas using the embryonic SC-like signature (C), the intestinal SC signature (D) and a modified embryonic SC-like signature that lack proliferation and *MYC* related genes **E**. **F**.-**H**. GSEA on human stage I colon carcinomas and restricted stage I *KRAS^wt^* and *KRAS^mut^* subsets that were stratified to *LGR5* status using the intestinal SC signature. **I**.-**J**. GSEA on human stage I colon carcinomas stratified to *KRAS* status using the embryonic SC-like signature (I) and intestinal SC signature (J). ES, enrichment score; NES, normalized enrichment score; FDR, false discovery rate.

To identify biologically meaningful gene expression patterns associated with the transition from colon adenoma to stage I colon carcinoma, we performed gene set enrichment analysis (GSEA) on human colon tissues using 3995 signatures of chemical and genetic pathways. We compared coherent transcriptional changes at the signature-level to identify specific patterns that were otherwise too variable to discern when considering expression profiles of individual genes [17]. Surprisingly, we found a significantly higher enrichment of multiple embryonic SC signatures than WNT signatures in malignant stage I human colon carcinomas (n = 17) versus benign colon adenomas (n = 26) (Figure 1A). This association was observed reproducibly with multiple independent embryonic SC signatures of different complexity (68-380 genes) that have few embryonic SC genes in common, which confirmed the significance of our findin­­g in human colon cancer initiation (Figure 1B). Next, we compared the stem cell programs that define the embryonic and intestinal SC identities by using validated embryonic and intestinal SC signatures of 380 and 132 non-overlapping genes that were derived from their respective stem cells (Supplemental Table 1) [15, 18]. When stage I colon carcinomas were compared to colon adenomas, the embryonic SC-like signature had a significantly higher normalized enrichment score than the intestinal SC signature (Figure 1C and 1D). Similar result was obtained when we used an alternate colon SC signature that was derived from relatively purified human colon SC (Supplemental Figure 1A).

Because proliferative capacity and self-renewal are intrinsic properties of stem cell identity, we verified that the embryonic SC-like signature did not just reflect proliferative differences between colon adenoma and stage I colon carcinoma. We ran the analysis using a modified embryonic SC-like signature that lacked genes associated with proliferation and cell cycle regulation. The association between stage I colon carcinoma and the modified embryonic SC-like signature remained significant (Figure 1E). Our analysis suggests that the activation of the embryonic SC-like signature is not a proxy for the proliferative difference between both colon adenoma and stage I colon carcinoma.

### KRAS^mut^ activates the embryonic SC-like signature

Because LGR5 and KRAS^mut^ have been implicated in the expansion of putative colon stem cells and inhibition of terminal differentiation [19, 20], we correlated their status with respect to both the embryonic SC-like and the intestinal SC signatures in human colon tissues. Consistent with a prior study, we found that stage I colon carcinomas that expressed *LGR5* (*LGR5^+^*; n= 10) significantly associated with the intestinal SC signature when compared to those that did not express *LGR5* (*LGR5^−^*; n= 7) (Figure 1F and Supplemental Figure 1B) [21]. Notably, our analysis further revealed that the association between the *LGR5* expression and the intestinal SC signature was observed only in the context of *KRAS^wt^* but not *KRAS^mut^* colon tumors (Figure 1G and 1H). Next, we stratified the stage I colon carcinomas according to their *KRAS* status and found that *KRAS^mut^* (n = 7) instead of *KRAS^wt^* (n = 10) was associated significantly with the embryonic SC-like signature, but not the intestinal SC signature (Figure 1I and 1J; Supplemental Figure 1C). This association was observed regardless of *LGR5* expression, which is consistent with *KRAS^mut^* as a dominant oncogenic driver (Supplemental Figure 1D and 1E). The embryonic SC-like signature has been correlated with high tumor grade by comparing poorly differentiated to well differentiated non-colorectal epithelial tumors [15]. To eliminate tumor grade differences as a confounding factor, we focused our comparison between *KRAS^mut^* versus *KRAS^wt^* colon tumors to moderately differentiated tumor grade and confirmed that all *KRAS^mut^* stage I colon carcinomas were of moderate differentiation. These findings suggest that the activation of the embryonic SC-like signature underlying the transition from colon adenoma to stage I colon carcinoma is correlated with *KRAS^mut^* expression and independent of tumor grade.

**Figure 2:**
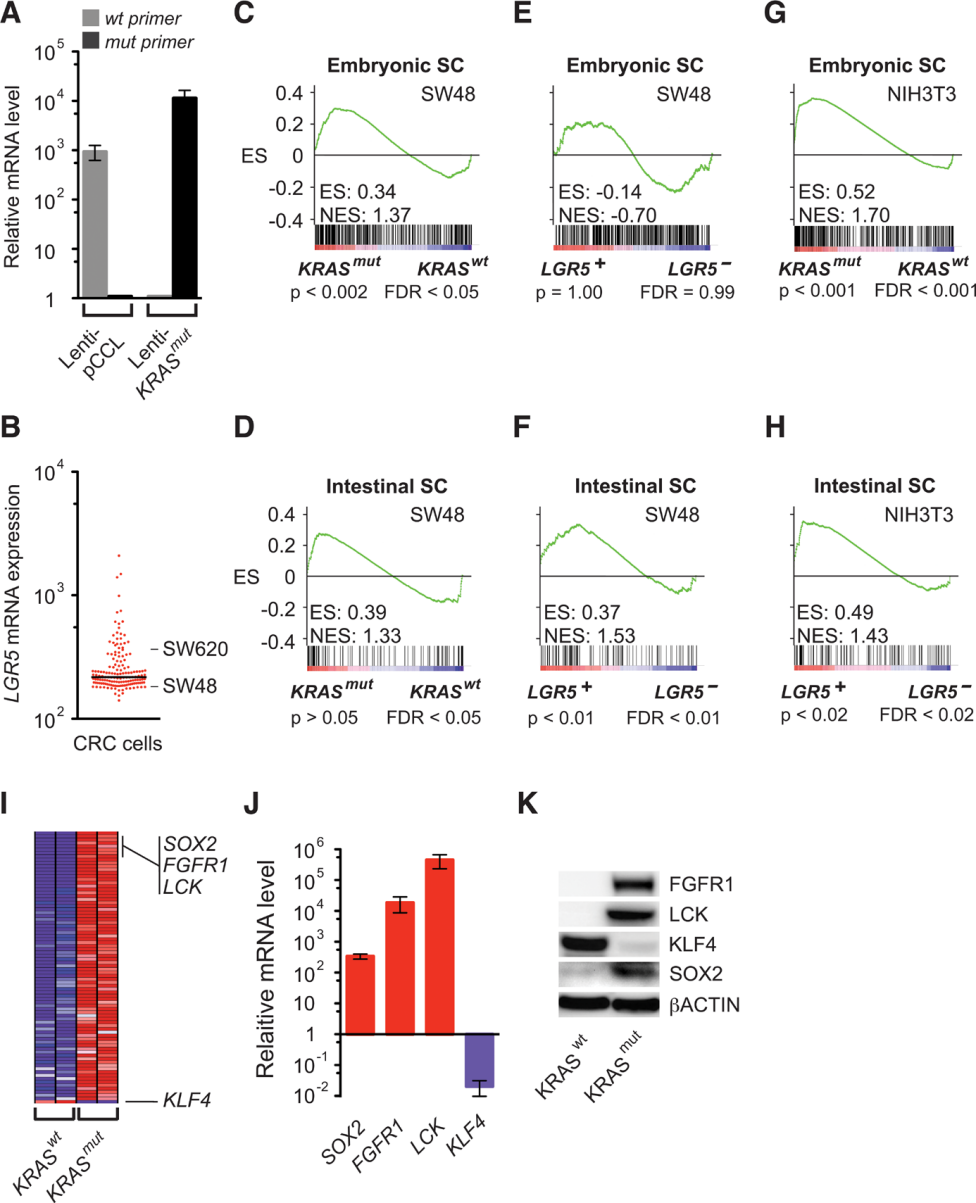
Activation of the embryonic SC-like and intestinal SC programs by *KRAS^mut^* and LGR5 **A**. Relative *KRAS* mRNA levels as measured by quantitative RT-PCR analysis using specific *KRAS^wt^* and *KRAS^mut^* primers in SW48 cells transduced with lenti-pCCL vector control or lenti-*KRAS^mut^*. **B**. Relative *LGR5* mRNA levels based on the gene expression profiles of 155 human colorectal cancer cells (GEO GSE59857). **C**.-**F**. GSEA using the embryonic SC-like (C, E) and intestinal SC (D, F) signatures on SW48 cells expressing lenti-pCCL vector control compared to lenti-*KRAS^mut^* (C, D) or lenti-*LGR5* (E, F). **G**. and **H**. GSEA using the embryonic SC-like and intestinal SC signatures on NIH3T3 cells expressing lenti-pCCL vector control compared to lenti-*KRAS^mut^* (G) or lenti-*LGR5* (H). **I**. H, Heat map of top 80 GSEA leading edge genes and *KLF4* in SW48 cells expressing lenti-*KRAS^mut^* and lenti-pCCL control (*KRAS^wt^*). Red, pink, light blue, and dark blue colors represent high, moderate, low, and lowest expression. I, Relative gene expression levels of *SOX2*, *FGFR1*, *LCK* and *KLF4* in SW48 cells expressing lenti-pCCL control versus lenti-*KRAS^mut^* as measured by quantitative RT-PCR analysis. **J**. Western blot of FGFR1, LCK, KLF4, SOX2 and βACTIN in SW48 cells expressing lenti-pCCL control versus lenti-*KRAS^mut^*.

We then addressed the challenge of analyzing complex behaviors in human tumors that consisted of different cell types by confirming the above findings in moderately differentiated SW48 human colon cancer cells. To determine if they directly induce distinct embryonic and intestinal SC programs, we expressed *KRAS^mut^*, *LGR5,* and pCCL control lentiviral vectors in SW48 cells, which harbored *KRAS^wt^* and had *LGR5* expression in the lower quartile of human colorectal cancer cells (Figure 2A and 2B). GSEA demonstrated that lenti-*KRAS^mut^* significantly activated the embryonic SC-like program, but not an intestinal SC program (Figure 2C and 2D). In contrast, SW48 cells expressing lenti-*LGR5* showed significant activation of the intestinal SC program, but not the embryonic SC-like program (Figure 2E and 2F). Thus *LGR5*, in addition to being a specific marker of intestinal SC, also enhanced the intestinal self-renewal program consistent with its potentiation of WNT signaling [22]. Interestingly, lenti-*KRAS^mut^* and lenti-*LGR5* expression in NIH3T3 mouse embryo fibroblasts also significantly activated the embryonic SC-like and intestinal SC programs, respectively (Figure 2G and 2H). Here, our findings in homogeneous SW48 cells reproduced those observed in human colon tissues, thus indicating that the activation of the embryonic SC-like and intestinal SC programs in human tissues was attributed to *KRAS^mut^* and *LGR5*, respectively.

The top genes that contributed most to the GSEA enrichment score are core factors enriched by *KRAS^mut^* within the embryonic SC-like program (Figure 2I and Supplemental Table 2). Several of these top leading-edge genes are known to activate reprogramming, including *FGFR1*, which plays a common role in both embryonic and cancer development [23], *LCK*, whose transcriptional silencing is required for ES cell differentiation [24], and the induced-pluripotency factor *SOX2*, which reprograms differentiated cells to pluripotency and is upregulated in colon cancer [25]. We validated that *SOX2, FGFR1* and *LCK* were highly upregulated at the mRNA and protein levels by lenti-*KRAS^mut^* expression (Figure 2J and 2K). contrast *KLF4* expression was suppressed in *KRAS^mut^* in colon cancer cells (Figure 2I-2K), which is consistent with its induction of multiple cell lineage differentiation in the intestine [26]. Overall, the expressions of these stem cell factors changed in accordance with an intestinal de-differentiation process.

### LGR5 promotes malignant transformation

**Figure 3:**
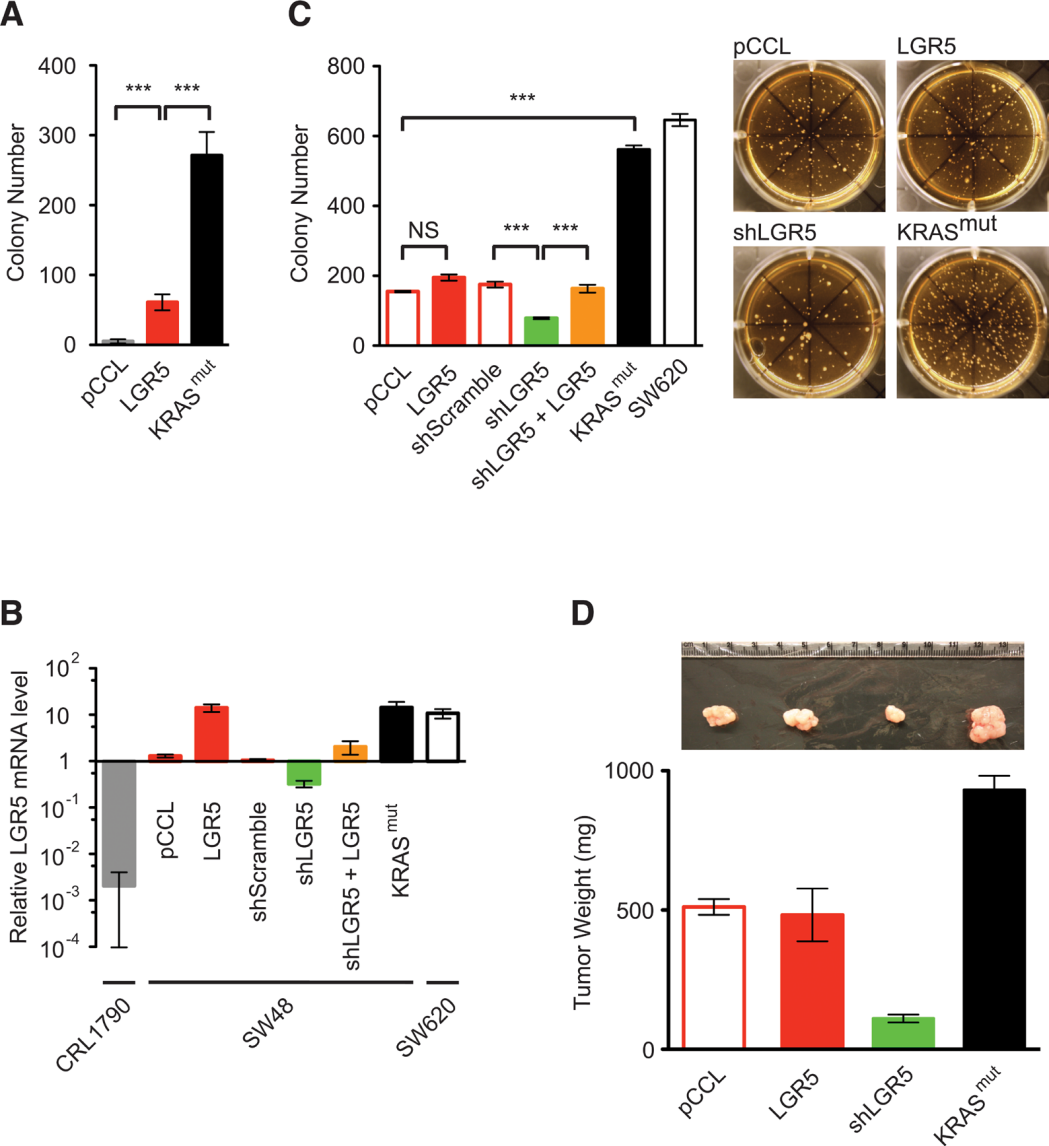
*KRAS^mut^* and LGR5 increase malignant transformation **A**. Soft agar colony growth assays of NIH3T3 cells expressing lenti-pCCL control, lenti-*LGR5* and lenti-*KRAS^mut^*. **B**. Relative *LGR5* gene expression levels of CRL1790, SW48 cells that had been modified by the indicated lentiviral transduction and SW620 as measured by quantitative RT-PCR analysis and normalized to parental SW48 cells. **C**. Soft agar colony growth assays of modified SW48 cells and SW620 cells with representative colony images (right). D. Tumor xenografts of modified SW48 cells with representative tumor images (upper). (***, p < 0.001). Quantitative data were averaged from n ≥ 3 independent experiments.

Cancer cells demonstrate anchorage-independent and clonogenic growth from a single cell, as measured *in vitro* by their ability to grow into colonies in soft agar growth medium [27]. Like cancer cells, LGR5^+^ SC are capable of anchorage-independent and clonogenic growth, yet the malignant potential conferred by LGR5 *per se* remains controversial [20, 28, 29]. Lenti-*LGR5* expression in NIH3T3 cells gave rise to limited soft agar colony growth when compared to respective negative and positive controls with lenti-pCCL and lenti-*KRAS^mut^* (Figure 3A). In SW48 cells, which had low endogenous *LGR5* expression when compared to non-expressing CRL1790 human colon differentiated epithelial cells and high expressing SW620 cells (Figure 2B and 3B), lenti-*LGR5* increased *LGR5* expression by 14.1 fold but did not enhance soft agar colony growth significantly when compared to lenti-pCCL control (Figure 3B and 3C). ShRNA suppression of endogenous *LGR5* expression in SW48 cells inhibited soft agar colony growth, an effect that was rescued by lentiviral re-expression of *LGR5* (Figure 3B and 3C). In comparison to *KRAS^wt^* SW48 cells, lenti-*KRAS^mut^* expression demonstrated significantly greater soft agar colony growth by > 2.5-fold, a malignant activity similar to SW620 cells, which expressed endogenous *KRAS^mut^* (Figure 3C). *In vivo* tumor growth of these modified SW48 cells in immunocompromized mice corresponded with their malignant growth in soft agar (Figure 3D). Although SW48-*KRAS^mut^* and SW620 cells also had increased *LGR5* expression (Figure 3B), *KRAS^mut^* was dominant in activating the embryonic SC-like program. Here, we found that LGR5, by imposing on cells an intestinal SC program, was required but not sufficient for efficient malignant transformation, which was optimized with *KRAS^mut^* activation of the embryonic SC-like program.

### Embryonic SC inhibitor suppressed KRAS^mut^ colon tumor growth

**Figure 4:**
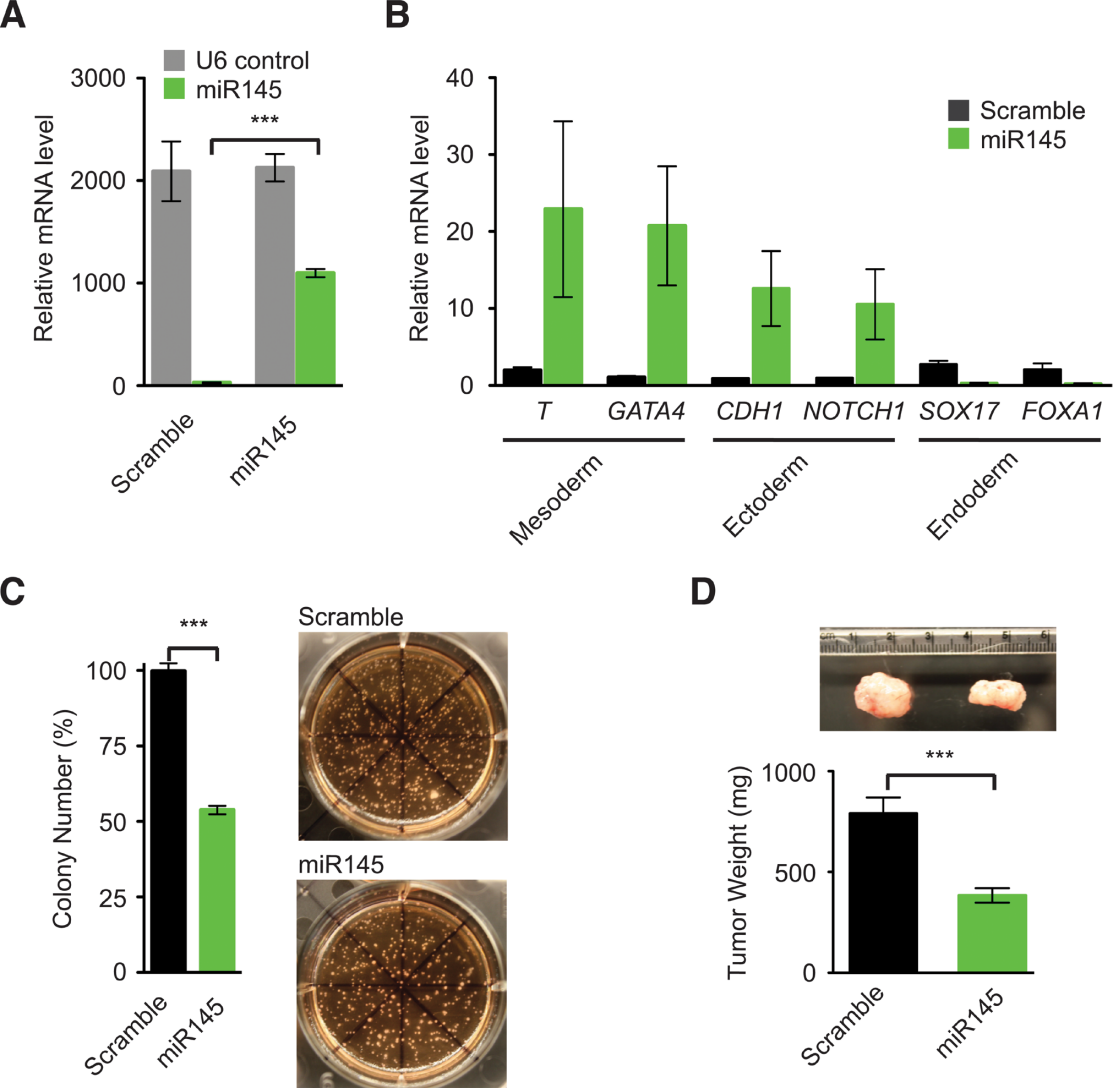
miR145 promotes gene expression of differentiation markers in *KRAS^mut^* colon cancer cells and inhibits tumorigenesis **A**. Relative U6 and miR145 levels in *KRAS^mut^* SW48 cells expressing lenti-pCCL-Scramble and lenti-pCCL-miR145 as measured by quantitative RT-PCR and normalized to parental SW48 cells. **B**. Relative gene expression levels of differentiation marker genes in *KRAS^mut^* SW48 cells expressing lenti-pCCL-Scramble or lenti-pCCL-miR145 as quantified by RT-PCR and normalized to parental *KRAS^mut^* SW48 cells. Brachyury (*T*), transcription factor GATA4, (*GATA4*), Notch homolog 1 translocation-associated (Notch1), E-cadherin (*CDH1*). **C**. Colony Growth of *KRAS^mut^* SW48 cells expressing lenti-pCCL-Scramble or lenti-pCCL-miR145 in soft agar assays with representative images (right). **D**. Tumor xenografts of *KRAS^mut^* SW48 cells expressing lenti-pCCL-Scramble and lenti-pCCL-miR145 with representative tumor images (upper). (***, p < 0.001). Quantitative data were averaged from n ≥ 3 independent experiments.

We hypothesized that activation of the embryonic SC-like program by KRAS^mut^ might underlie its tumorigenicity, and suppression of the embryonic SC-like program should decrease the malignant potential conferred by KRAS^mut^. As systemic regulators of biological processes, some microRNAs (miRNA) are known to regulate embryonic SC pluripotency and differentiation [30]. In particular, miR145 was demonstrated to induce human embryonic SC lineage-restricted differentiation through direct inhibition of pluripotency genes and suppression of human embryonic SC self-renewal [31]. Just like human embryonic SC, human colon tumors and the parental *KRAS^wt^* SW48 cells, *KRAS^mut^* SW48 cells, which harbor the embryonic SC-like program, did not express miR145 (Figure 4A) [31, 32]. In comparison to lenti-Scramble control, transduction of lenti-miR145 into *KRAS^mut^* SW48 cells increased the relative gene expression of well-known mesoderm and ectoderm differentiation markers (Figure 4A and 4B). Because colon cancer cells are of endodermal origin, *KRAS^mut^* SW48 cells had high basal expression of endoderm differentiation markers that were not increased further by miR145 expression (Figure 4B). To test the ability of miR145 to inhibit tumorigenic growth, we performed both *in vitro* soft agar growth assays and *in vivo* tumor xenograft experiments in immunocompromised mice. The expression of lenti-miR145 in *KRAS^mut^* SW48 cells reduced their clonogenic growth in soft agar by 43% (Figure 4C). Furthermore, miR145 inhibited the tumor growth of *KRAS^mut^* SW48 cells by 51% (Figure 4D). These data suggest that anchorage-independent and clonogenic malignant growth is more efficiently acquired through the *KRAS^mut^* mediated embryonic SC-like program, which is dominant even in SW48 cells that initially expressed LGR5 and the intestinal SC program.

### Spatiotemporal *LGR5* expression in human colon cancer development

**Figure 5:**
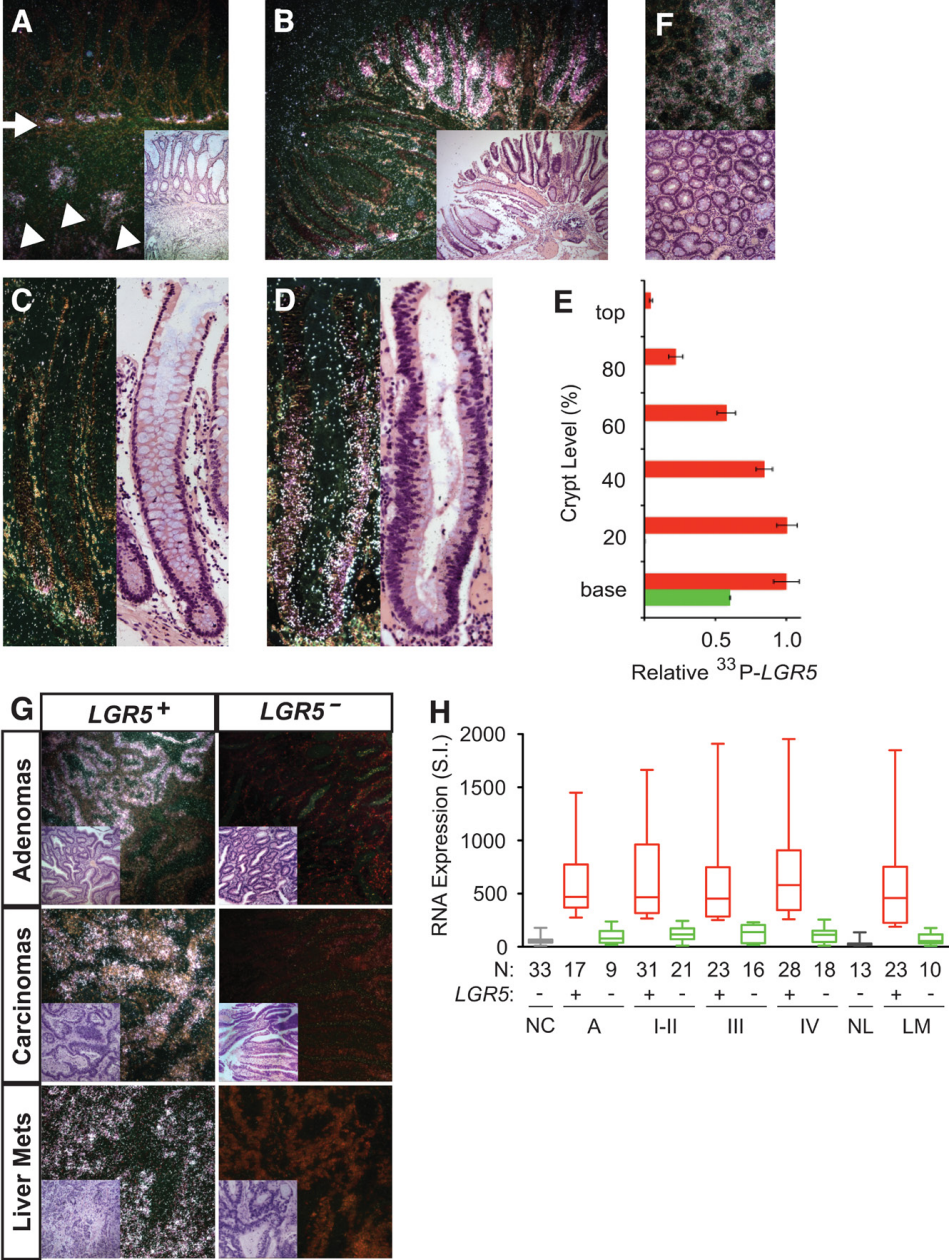
Spatiotemporal *LGR5* expression in human colon cancer Representative dark field images of *in situ* hybridization with ^33^P-probe specific for human *LGR5* mRNA and their corresponding H&E stainings in human colon adenomas and colon carcinomas. **A**. *LGR5* mRNA expression in invading colon cancer cells (white arrowhead) and the adjacent hyperplastic crypt base stem cells (white arrow) of a primary colon carcinoma. **B**.-**D**. Representative *LGR5* mRNA expression in hyperplastic (B, C) and aberrant (B, D) crypts of human colon adenomas. **E**. Relative *LGR5* expression was quantified according to crypt level in 25 hyperplastic crypts (green bar) and 25 dysplastic crypts (red bar) of human colon adenomas. **F**. Axial cross-sections of dysplastic crypts of a human adenoma showed crypt clustered *LGR5* expression. **G**. Representative dark field images of *in situ* hybridization with ^33^P-probe specific for human *LGR5* mRNA in colon adenomas, primary colon carcinomas, and colon liver metastases (H&E inset). **H**. Turkey-whisker plot of *LGR5* gene expression in human mucosal tissues of normal colon (NC), colon adenomas (Ad), colon primary carcinomas (I-IV), normal liver (NL), and colon metastases to the liver and lung (LM). Patient numbers (N) are as indicated.

Since LGR5 promotes malignant transformation and increases the intestinal SC gene signature, LGR5^+^ colon SC is likely the predominant adenoma-initiating cell for the development of a human colon adenoma. Lineage tracing studies have shown that murine adenomas can originate from crypt base SC origin, since targeted *APC* deletion in murine LGR5^+^ intestinal SC gave rise to adenomas [12]. To identify human colon adenoma-initiating cells, we first performed *LGR5 in situ* hybridization to examine the spatiotemporal pattern of LGR5^+^ colon SC during human colon adenoma initiation. ^33^P-*LGR5 in situ* signals in adjacent benign hyperplastic colon crypts were restricted to their crypt base origin in contrast to their diffuse invasiveness in primary human LGR5^+^ colon carcinomas (Figure 5A and Supplemental Figure 1H). To clarify the spread of ^33^P-*LGR5 in situ* signals, we labeled human colon adenomas that contained intact hyperplastic crypts with benign colon cells and adjacent aberrant crypts that had dysplastic cells with hyperchromatic and enlarged nuclei throughout the entire crypt axis (Figure 5B-5D). Like those in human colon carcinomas, hyperplastic crypts in colon adenomas had ^33^P-*LGR5 in situ* signals entire­ly at the crypt bases (Figure 5B, 5C, 5E). However, all aberrant crypts had a ^33^P-*LGR5 in situ* signal pattern of maximal expression at the crypt bases with gradual absence of *LGR5* expression in dysplastic cells at varying levels toward the crypt tops (Figure 5B, 5D, 5E). We did not observe the inverse pattern of greater ^33^P-*LGR5 in situ* signals higher up the crypt axes, or the absence of crypt base signals. Cross-sections perpendicular to the crypt axes of colon adenomas revealed *LGR5^+^* and *LGR5^−^* crypt clustered patterns but not a hybrid *LGR5^+^*/*LGR5^−^* crypt cluster pattern that may arise from stochastic *LGR5* re-expression towards the crypt top (Figure 5F). Overall, these spatiotemporal *LGR5* patterns are consistent with a crypt base model of colon adenoma initiation.

We next examined the patterns of *LGR5* expression in human colon adenomas, carcinomas and distant metastases. Outgrowth of *LGR5^+^* and *LGR5^−^* dysplastic cells beyond the colon crypt structures gave rise to either hybrid *LGR5^+^*/*LGR5^−^* or *LGR5^−^* colon adenomas (Figure 5G). Malignant transformation of colon adenomas into primary colon carcinomas and liver metastases resulted in two distinct patterns of *LGR5^+^* or *LGR5^−^ in situ* signals throughout the malignant epithelia and not the hybrid *LGR5^+^*/*LGR5^−^* pattern (Figure 5G). Quantification of *LGR5* gene expression in a large cohort of 26 colon adenomas, 137 primary colon carcinomas and 33 liver and lung metastases revealed that approximately two third had *LGR5^+^* patterns and one third had *LGR5^−^* patterns compared to normal colon and liver controls (Figure 5H). Median and mean *LGR5* gene expression in *LGR5^+^* colon adenomas, carcinomas and liver or lung metastases were 5-10 fold higher than those in normal controls and *LGR5^−^* tissues (Figure 5H; Supplemental Table 3). These findings suggest that while human colon adenomas are derived predominantly from *LGR5^+^* colon crypt cells, human colon carcinomas may originate from both *LGR5^+^* and *LGR5^−^* adenoma cells.

### Activation of the embryonic SC-like and EMT programs in stage I *LGR5^−^* colon carcinoma

**Figure 6:**
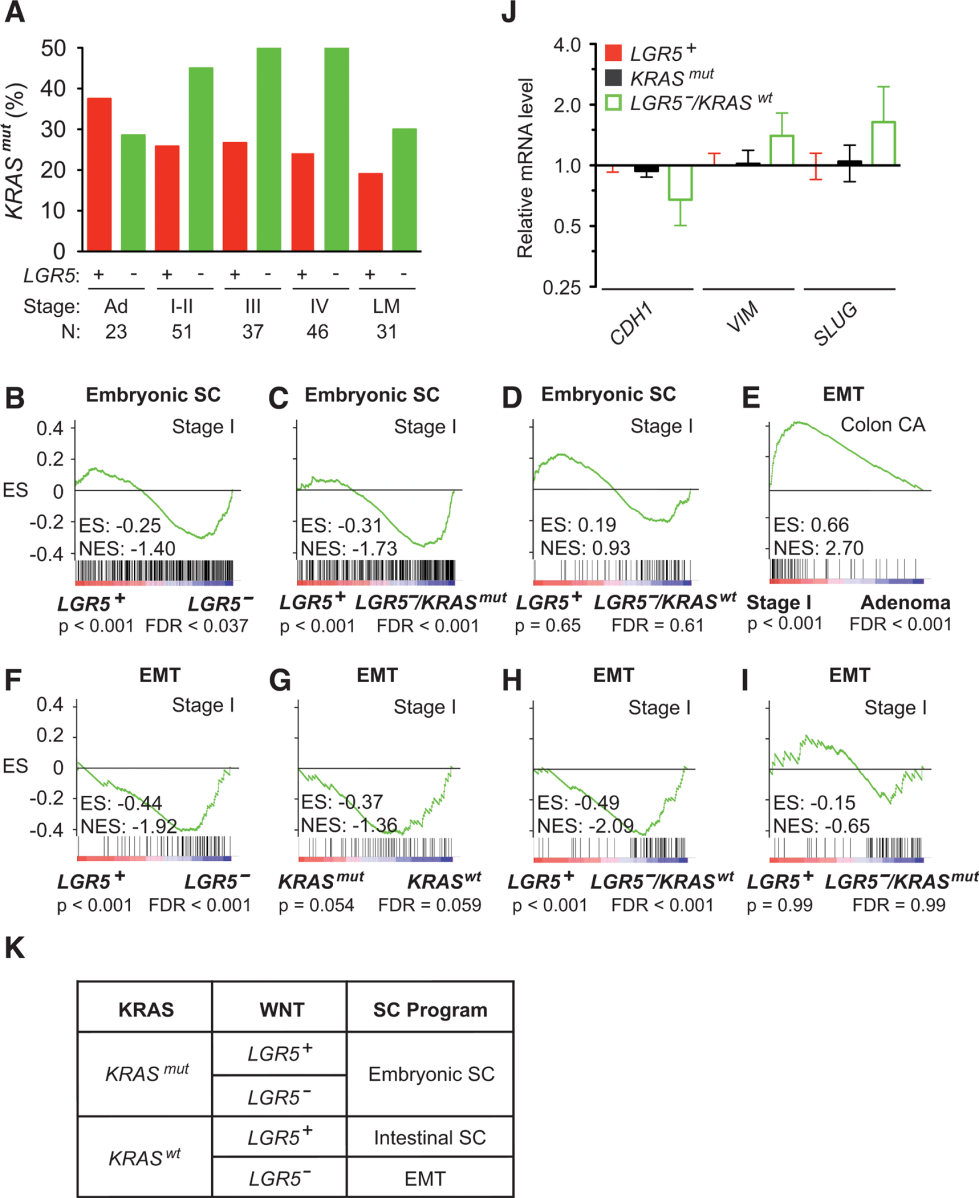
Activation of the embryonic SC-like and EMT programs in *LGR5^−^* colon carcinoma **A**. Frequency of *KRAS^mut^* status with respect to *LGR5* status in human stage I to IV colon carcinomas. Patient numbers (N) are as indicated. **B**.-**D**. GSEA on human stage I colon carcinomas using the embryonic SC-like signature stratified to *LGR5* status (B) and combined *LGR5* and *KRAS* status (C, D). E. GSEA using the EMT signature on human stage I colon carcinomas versus colon adenomas. **F**.-**I**. GSEA using the EMT signature on human stage I colon carcinomas stratified to *LGR5* (F)*, KRAS* (G) and combined *LGR5/KRAS* (H, I) status. ES, enrichment score; NES, normalized enrichment score; FDR, false discovery rate. **J**. Relative mRNA levels of EMT-related markers in *LGR5^+^*, *KRAS^mut^* and *LGR5^−^/KRAS^mut^* stage I colon carcinomas. **K**. Schematic classification of the embryonic SC-like, intestinal SC and EMT programs during stage I colon cancer initiation based on *KRAS^mut^* and *LGR5* status.

As LGR5 promotes malignant transformation, its absence in *LGR5^−^* carcinomas suggests the presence of another oncogenic driver. The frequency of *KRAS^mut^* increases as colon adenomas enlarge and become more predisposed to malignant transformation into colon carcinomas [2]. Strikingly, we observed in the transition from colon adenomas (n = 23) to stage I-IV colon carcinomas (n= 165) that *LGR5^−^* status was associated with a 1.9 fold higher frequency of *KRAS^mut^* status (Figure 6A). This suggests that colon cancer initiation from *LGR5^−^* adenomas into carcinomas selected for the presence of *KRAS^mut^*. GSEA of stage I colon carcinomas showed significant activation of the embryonic SC-like program in *LGR5^−^* compared to *LGR5^+^* tumors (Figure 6B). This activation of the embryonic SC-like program was attributed to *KRAS^mut^*, but not *KRAS^wt^* status (Figure 6C and 6D).

Since the initiation of *LGR5^−^/KRAS^wt^* stage I colon carcinoma did not activate the embryonic SC-like or intestinal SC program (Supplemental Figure 1F and 1G), we assessed by GSEA whether the de-differentiation program of epithelial to mesenchymal transition (EMT) was involved. In comparison to colon adenomas, stage I colon carcinomas significantly enriched for the consensus EMT program (Figure 6E) [33]. Initial stratification of stage I colon carcinomas based on either *LGR5* or *KRAS* status showed activation of the EMT program in those with *LGR5^−^* or *KRAS^wt^* status (Figure 6F and 6G). Furthermore the EMT program was activated in *LGR5^−^/KRAS^wt^* but not *LGR5^−^/KRAS^mut^* colon carcinomas (Figure 6H and 6I). We confirmed that relative to those with *LGR5^+^* and *KRAS^mut^* status, *LGR5^−^/KRAS^wt^* colon carcinomas had decreased *CDH1* and increased *VIM* and *SLUG* expressions, which are the expected changes in EMT biomarkers (Figure 6J). Thus, the initiation of *LGR5^−^* stage I colon carcinoma is associated with the activation of the embryonic SC-like program in *KRAS^mut^* tumors and the EMT program in *KRAS^wt^* tumors (Figure 6K).

## DISCUSSION

By using an integrated systems-level approach to analyze genetic, clinical and translational data, we elucidated the significance of distinct stem cell programs during human colon cancer initiation. Aberrant expression of LGR5, a WNT target gene that potentiates WNT signaling [34], induced the intestinal SC program and contributed to moderate malignant potential in the context of *KRAS^wt^* cells. This is consistent with the finding that targeted *APC* deletion in murine LGR5^+^ colon SC gave rise to adenomas [12]. In an alternate murine intestinal tumor model, differentiated colon epithelial cells with *LGR5^−^/KRAS^wt^* status demonstrated the potential to dedifferentiate into LGR5^+^ adenoma-initiating cells when KRAS^mut^ and transcription factor NF-κB were activated to enforce high Wnt signaling [35]. Both of these studies suggest that constitutive activation of the intestinal SC program is sufficient for rapid and efficient malignant transformation of murine LGR5^+^ colon stem cells and LGR5^−^ differentiated colon cells into murine adenomas. Human colon adenomas also arise from WNT activation of the intestinal SC program [36]. However, human cells require a much higher threshold for malignant transformation than murine cells, which are more readily transformed [37]. Left untreated, the natural history of human colon adenomas is one of slow progression to colon carcinomas over a decade. This slow process may reflect the moderate malignant potential of *LGR5^+^*/*KRAS^wt^* colon carcinomas, which are driven primarily by the intestinal SC program, and the longer period of time needed to acquire a more aggressive oncogene such as *KRAS^mut^*.

We demonstrated that the malignant transformation of a pre-malignant adenoma to stage I colon carcinoma by *KRAS^mut^* preferentially activated the embryonic SC-like program instead of the intestinal SC program. Although *KRAS^mut^* may also enhance certain aspects of WNT signaling [38], *KRAS^mut^* was dominant in activating the embryonic SC-like program regardless of LGR5 expression. We have linked the activation of the embryonic SC-like program in *KRAS^mut^* colon cancer cells with its potential for malignant transformation by showing that miR145, a well established repressor of embryonic SC development [31], increased the expression of differentiation markers and suppressed their ability to initiate tumors. Since the embryonic SC-like program does not exist a priori in any colon cell, we have identified a specific dedifferentiation program that greatly enhances their malignant transformation. It was recently reported that *KRAS^mut^* confers a significant competitive advantage to LGR5^+^ intestinal SC over their *KRAS^wt^* SC neighbors [38-40]. However, *KRAS^mut^* SC are not deterministically fixed but instead are, to a certain extent, stochastically replaced by *KRAS^wt^* SC, thus indicating that *KRAS^mut^* can also be lost within the SC population. It was suggested that the biased stochastic advantage conferred by *KRAS^mut^* on intestinal SC clonal expansion may be attributed mainly to its stimulation of proliferation. Proliferative capacity and self-renewal are quintessential properties of stem cell identity, and genes associated with cell cycle and proliferation such as *MYC* and its related targets genes are included in the embryonic SC-like program [41]. Notably, we still observed a significant enrichment for the modified embryonic SC-like program that excluded genes associated with cell proliferation and *MYC*-related target genes. Therefore, the bias competitive advantage provided by *KRAS^mut^* on clonal expansion was attributed to its activation of the embryonic SC-like program and not just proliferation.

In the absence of *KRAS^mut^* and *LGR5* oncogenic drivers, environmental factors such as chronic inflammation may act to drive colon cancer initiation. The risk for developing colorectal cancer increases with higher severity and duration of chronic inflammation in ulcerative colitis and Crohn's disease [42]. In contrast to sporadic colorectal carcinomas, in which the majority harbors *APC*, *CTNNB1* or *KRAS* mutations, colorectal carcinomas that arise in the setting of chronic inflammatory diseases have a very low frequency of these mutations [43, 44]. While EMT has been implicated as an alternate oncogenic pathway in colorectal cancer progression and metastases [33], EMT also contributes to the pathogenesis of ulcerative colitis and Crohn's disease [45, 46]. Here we demonstrated that *LGR5^−^*/*KRAS^wt^* stage I colon carcinomas activated the EMT program, which reveals an earlier role by EMT in the initiation of colorectal cancer induced by inflammation.

The critical transition from a pre-malignant adenoma to stage I colon carcinoma is defined pathologically by the ability of malignant cells to invade the colonic submucosa. These newly transformed cancer-initiating cells thrive due to their ability to adapt to the new tumor environment. Since the embryonic SC program is associated with pluripotency, we expect colon cancer-initiating cells that express the embryonic SC-like program to have high plasticity that imparts greater phenotypic flexibility to adapt to changes in the tumor environment, which provides a competitive survival advantage. The EMT program also endows a high degree of plasticity and is defined by a reversible developmental program that allows the transition between phenotypes with silencing of epithelial differentiation genes and re-expression of mesenchymal genes [47]. In contrast, the intestinal SC program is associated with multipotency and moderate plasticity, and it controls multilineage intestinal tissue differentiation. The degree of plasticity determines the dynamic ability of a cell to adopt a different developmental gene program that regulates cellular differentiation. Under pathological conditions, plasticity increases due to dedifferentiation under oncogenic and/or environmental pressures and decreases as a cell differentiate. We have identified high cellular plasticity beyond the intestinal SC program as a key malignant transformation property of human colon cancer-initiating cells. Although *KRAS^mut^* did not induce pluriopotency *per se*, the greater plasticity associated with the embryonic SC-like program in comparison to the intestinal SC program contributed to its higher malignant potential. Interestingly, senescence markers from oncogene-induced senescence and mTOR-mediated geroconversion have been detected in pre-malignant lesions, including colon adenomas [48-51]. We suggest that *KRAS^mut^* induces malignant transformation by increasing the plasticity of colon cancer-initiating cells to bypass senescence.

**Figure 7:**
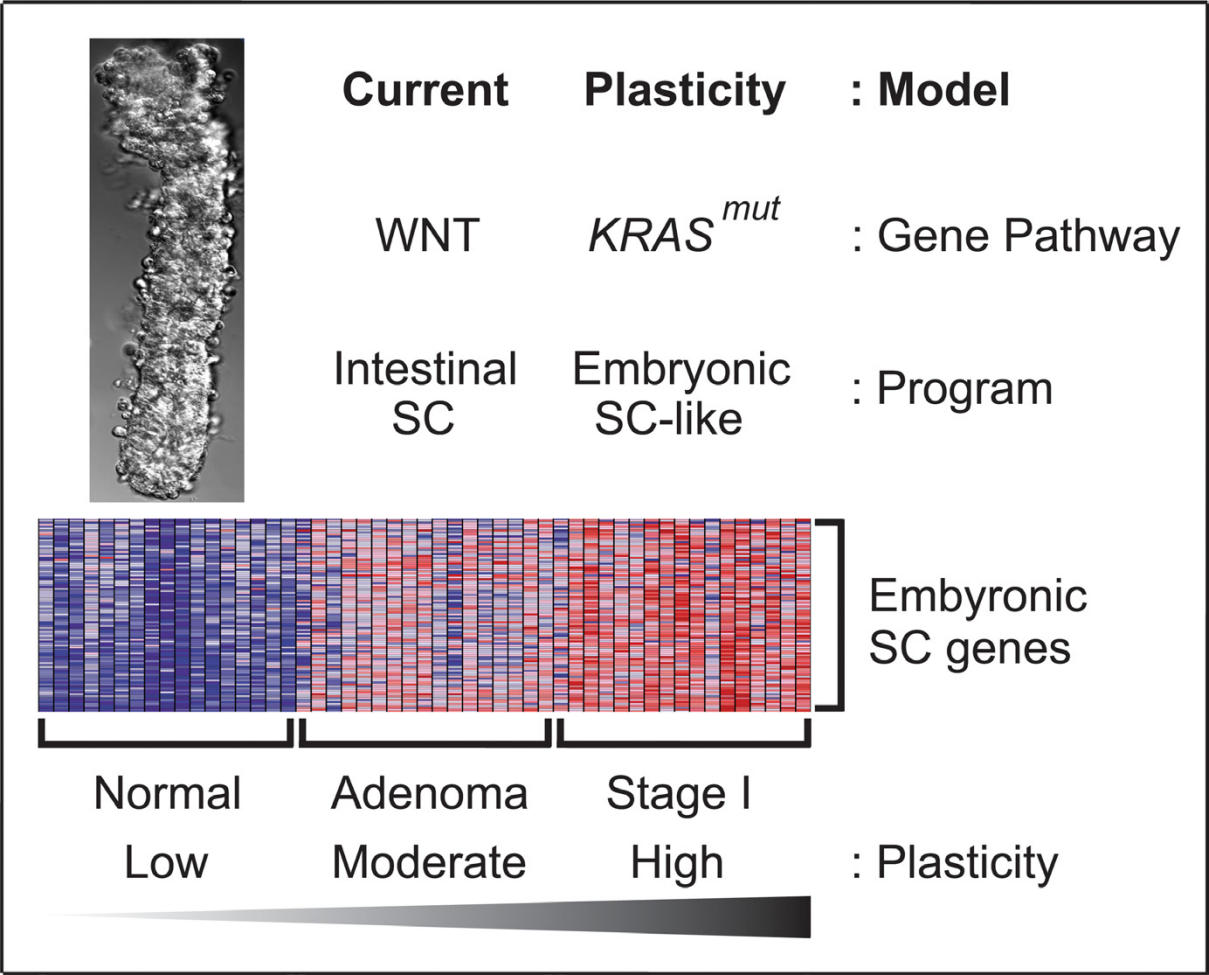
*In vivo* plasticity model of human colon cancer initiation *KRAS^mut^* induced high cellular plasticity with the embryonic-SC like program to transform human colon adenomas to carcinomas. Heatmap of selected embryonic SC genes showed low, moderate and high expressions in normal colon epithelia of colon crypt, colon adenomas and stage I colon carcinomas, respectively. Red, pink, light blue, and dark blue colors represent high, moderate, low, and lowest expression.

Based on our findings, we propose an *in vivo* plasticity model of human colon cancer initiation that merges high cellular plasticity with *KRAS^mut^* to optimize malignant transformation (Figure 7). First, *APC* or *CTNNB1* mutations in LGR5^+^ colon stem cells give rise to human colon adenomas by constitutively activating WNT signaling to sustain the intestinal SC program and bypass cellular differentiation. Within these plastic adenoma cells, *KRAS^mut^* activates the embryonic SC-like program and initiates the first stage of colon carcinoma development. In our model, the degree of plasticity of the adenoma-initiating cells undergoing the oncogenic pressure is advantageous because the LGR5^+^ colon stem cells exhibit enough inherent plasticity to adopt a developmental gene expression program and give rise to an aggressive tumor instead of normal tissue. The degree of plasticity of the colon adenoma-initiating cell lies not only in the intrinsic stem cell properties of the LGR5^+^ colon stem cell, but more importantly, is a direct consequence of *KRAS^mut^*, which induces dedifferentiation by activating the embryonic SC-like program beyond the intrinsic intestinal SC program of the LGR5^+^ colon stem cell. Our plasticity model of human colon cancer initiation complements and expands upon the current model based on constitutive WNT signaling and the intestinal SC program.

Therapeutic inhibition of *KRAS^mut^* is an unmet clinical need, especially because *KRAS^mut^* tumors have higher inherent chemotherapy resistance, do not respond to anti-EGFR targeting and are frequent drivers of acquired resistance in *KRAS^wt^* tumors treated with anti-EGFR therapy [6, 52]. Current strategies to inactivate *KRAS^mut^* involve targeting RAS based on its structural characteristics or well-established RAS effector pathways. Unfortunately, inhibitors that interfere with the post-translational farnesylation or geranylgeranylation of KRAS have shown limited clinical efficiency or excessive off-target toxicities [7]. The dual inhibition of the Raf-MEK-ERK and PI3K/AKT downstream pathways gave overlapping toxicities that precluded sufficient target inhibition to achieve clinical activity [9]. These narrowly focused therapeutic approaches may be bypassed by the complex redundant effectors that are active in *KRAS^mut^* colorectal cancer. Here, we pursued a systems-level approach by profiling gene expression patterns in human colon cancer tissues according to their *KRAS^mut^* status, and we have uncovered a global mechanism driving colon cancer initiation. Our discovery of KRAS^mut^-mediated activation of the embryonic SC-like program has promising translational implications. Global inhibition of the embryonic SC-like program, by epigenetic regulators such as miRNAs (e.g., mir145), or inhibitors of multiple key pathways involved in embryonic SC signaling represents novel therapeutic strategies for blocking *KRAS^mut^* colon tumor development. Despite shortcomings in their delivery efficiency, miRNAs have emerged as promising therapeutic agents because of their global effect on biological processes. We see two main advantages to the therapeutic inhibition of the embryonic SC-like program in colorectal cancer. First, we expect these embryonic SC inhibitors to have large therapeutic windows with minimal off-target toxicity since the embryonic SC-like program is activated in cancer cells, but not in both adult stem cells and differentiated cells. The fact that cancer incidence increases with age when there is a decreased physiologic role for embryonic SC signaling may also minimize the overall toxicity from this therapeutic strategy. Second, these inhibitors may be especially effective in colon cancer prevention given that activation of the embryonic SC-like program is an event that occurs very early in colon cancer development and not just in poorly differentiated cancers [15]. Currently, the prevention of colon adenoma transformation to carcinoma is best achieved physically by endoscopic resection. Overall, targeting of the embryonic SC-like program may be the Achilles heel of colorectal cancer initiation and *KRAS^mut^* tumors.

## MATERIALS AND METHODS

### Reagents, cells, mice and human tissues

Antibodies to SOX2 and KLF4 (R&D), FGFR1 and LCK (Cell Signalling), KRAS (CalBiochem) and β-ACTIN (Santa Cruz) were used in western blot based on published protocol. NIH3T3 mouse embryo fibroblasts, human SW48 and SW620 colon cancer cells, and human CRL1790 colon epithelial cells were cultured with DMEM and 10% fetal bovine serum for less than 6 months from expanded frozen stocks that were obtained initially from American Type Culture Collection (ATCC; Manassas, VA). Nude mice (Crl: NU-*Foxn1^nu^*) were obtained from Charles River. Murine research complied with UC Irvine Institutional Animal Care and Use Committee approved protocol. Human tissues were collected prospectively under Institutional Review Board protocol from patients undergoing elective surgery for colon cancer at Memorial Sloan-Kettering Cancer Center from January 1990 to December 2000. Tissues included normal colon, normal liver, colon adenomas, moderately differentiated primary colon carcinomas, and liver and lung metastases from primary colon cancer. Human tissues were snap-frozen in liquid nitrogen or embedded in Tissue-Tek optimal cutting temperature compound blocks and paraffin. Mucosal tissues were obtained by manual microdissection under microscopic visualization to minimize stromal contaminant.

### Plasmids and primers

Human LGR5 ORF was amplified by PCR from pCR-BluntII-TOPO-LGR5 vector (ATCC) using forward primer 5′-CTC GGA TCC ACC GCC ATG GAC ACC TCC CGG CTC GGT GT-3′ and reverse primer 5′-AGA GTC GAC TTA GAG ACA TGG GAC AAA TGC CAC AGA GG-3′ and cloned into lentiviral pCCL-PGK vector (lenti-pCCL) at the 5′BamHI and 3′ SalI sites of pCCL-PGK (pCCL-PGK-LGR5). BamHI and EcoRI restriction digest of pCCL-PGK-LGR5 gave a 0.7kb N-terminal LGR5 ORF fragment that was cloned into the corresponding sites of pBSSK^+^(pBSSK^+^LGR5^−^). *KRAS^12V^* ORF was amplified by PCR from pcDNA3.1^+^neo-KRAS^12V^ using forward primer 5′-GGG GGA TCC ACC GCC ATG ACT GAA TAT AAA CTT GTG-3′ and reverse primer 5′-GATT GTC GAC TTA CAT AAT TAC ACA CTT TGT CTT TGA C-3′ and cloned into the 5′BamHI and 3′SalI sites of pCCL-PGK (pCCL-PGK-*KRAS^12V^*). Overlapping primers for shRNA Scramble control and miR145 were cloned into the 5′BamH1 and 3′SalI sites of lentiviral pCCL-PGK vector. Primers for quantitative RT-PCR of U6 snRNA and miR145 (target sequence 5′-GUC CAG UUU UCC CAG GAA UCC CU-3′) were obtained from Exiqon. The primer sequences used for cloning and quantitative RT-PCR are as listed in the following Table 1.

**Table 1:** primer sequences

Gene	Sense Primer	Anti-sense Primer
KRASwt	5′TGG TAG TTG GAG CTG G3′	5′ttg ttg gat cat att cgt3′
KRASmut	5′TGG TAG TTG GAG CTG T3′	5′ttg ttg gat cat att cgt3′
LGR5	5′AAC CTT TAC CAG CTC CAG CA3′	5′ATG CCA CAG AGG AAA GAT GG3′
SOX2	5′CAA GAT GCA CAA CTC GGA GA3′	5′GCT TAG CCT CGT CGA TGA AC3′
FGFR1	5′AAC CTG ACC ACA GAA TTG GAG GCT3′	5′ATG CTG CCG TAC TCA TTC TCC ACA3′
LCK	5′CAC GAA GGT GGC GGT GAA GA3′	5′GAA GGG GTC TTG AGA AAA TCC A3′
KLF4	5′CCA ATT ACC CAT CCT TCC TG3′	5′CGA TCG TCT TCC CCT CTT TG3′
BRACH-YURY (T)	5′CCG TCT CCT TCA GCA AAG TC3′	5′CAC CGC TAT GAA CTG GGT CT3′
GATA4	5′TCC AAA CCA GAA AAC GGA AG3′	5′TGC CCG TAG TGA GAT GAC AG3′
CDH1	5′GAA CGC ATT GCC ACA TAC AC3′	5′ATT CGG GCT TGT TGT CAT TC3′
NOTCH1	5′GGG CTT CAA AGT GTC TGA GG3′	5′CGG AAC TTC TTG GTC TCC AG3′
SOX17	5′GGG GAC ATG AAG GTG AAG G3′	5′TTG TGC AGG TCT GGA TTC TG3′
FOXA1	5′CCG TTC TCC ATC AAC AAC CT3′	5′GAG CCG TAA GGC GAG TAT TG3′
B2M	5′AGG CTA TCC AGC GTA CTC CA3′	5′TCA ATG TCG GAT GGA TGA AA3′
Lenti-miR145	5′GAT CTA CTA GTC ACC TTG TCC TCA CGG TCC AGT TTT CCC AGG AAT CCC TTA GAT GCT AAG ATG GGG ATT CCT GGA AAT ACT GTT CTT GAG GTC ATG GTT AT3′	5′TCG AAT ATA ACC ATG ACC TCA AGA ACA GTA TTT CCA GGA ATC CCC ATC TTA GCA TCT AAG GGA TTC CTG GGA AAA CTG GAC CGT GAG GAC AAG GTG ACT AGT A3′
Lenti-Scramble	5′GAT CCC CGC TTG TTC GTT GGT AAC TAC ATT CAA GAG ATG TAG TTA CCA ACG AAC AAG CTT TTT A3′	5′TCG ATA AAA AGC TTG TTC GTT GGT AAC TAC ATC TCT TGA ATG TAG TTA CCA ACG AAC AAG CGG G3′

### Generation of lentiviruses

Lentiviruses were generated by cotransfecting 293T cells with 15 μg of lentiviral vectors (pCCL, pCCL-GFP, pCCL-Lgr5, pCCL-Kras12V, pCCL-shScramble and pCCL-miR145), 3.5 μg of pENV/VSV-G, 6 μg of pRRE, and 3 μg of pRSV-REV in 293T cells using BioT (Bioland Scientific) per manufacturer instruction. Supernatants were collected on days 3 and 4 post-transfection. Cells were transduced with lentivirus at MOI=10 and 6μg/ml polybrene (Sigma) and maintained in DMEM growth medium.

### Soft agar colon growth and murine xenograft tumor assays

NIH3T3 cells (10^4^) and human colon cancer cells (10^3^) were grown in growth media with a 0.33% top layer and a 0.5% bottom layer that included Difco Bacto Agar (w/v) in 6 well plates. Colonies greater than 50 cells were counted visually using a stereo zoom microscope at 3 weeks and imaged at 6 weeks. Human colon cancer cells (5×10^6^) were injected into the dorsal subcutaneous flank of *Foxn1^nu^*/*Foxn1^nu^* Nude mice. The growth of tumor xenografts were assessed at 21 days. All mouse experiments were done in compliance with UCI and Istitutional Animal Care and Use Committee (IACUC) policies.

### *In situ* hybridization

RNA *in situ* hybridization was performed mainly as described [53]. Briefly, paraffin tissue sections 5 μm thick were cut and mounted on Fisher Superfrost Plus slides. ^33^P-UTP-labeled antisense and sense 0.7kb probes were generated from EcoRI and BamHI linearized pBSSK^+^LGR5 with T3 and T7 RNA polymerases, respectively, in the presence of 12 μM cold UTP and 4μM ^33^P-UTP. The sense probe served as a negative control. The slides were rehydrated, fixed in 4% paraformaldehyde and treated with proteinase K prior to deacetylation. Slides were prehybridized at 65°C for 3 h in hybridization buffer containing 50% formamide and 1x SSC (0.15 M NaCl and 0.015 M sodium citrate) prior to overnight hybridization with radiaoactive probe at 3 × 10^6^ cpm/slide. The washes included 5x SSC at 55°C for 10 min, 50% formamide and 1x SSC at 65°C for 20 min, twice with RNAase buffer (0.3 M NaCl, 10 mM Tris pH 8.0, and 5 mM EDTA) at 37°C for 30 min, RNase buffer with 50 μg/ml RNase A at 37°C for 15 min, and twice in 2x SSC and 0.1x SSC at 25°C for 15 min each. The slides were dehydrated with ethanol and dipped in autoradiographic emulsion (NTB-2; Kodak). After 2 months of exposure, the slides were developed in Kodak developer D-19, fixed in Kodak fixer, and counterstained with hematoxylin and eosin. To determine relative *LGR5* expression along the hyperplastic or dysplastic crypt axis level, each crypt was divided into five levels along its axis (0-20%, 20-40%, 40-60%, 60-80% and 80-100%). Silver grains were quantified at each level and normalized relative to that of the corresponding crypt base, which was assigned a value of 1.

### Microarray gene expression profile

Total RNA was purified with Qiagen RNeasy purification column and reagents (Invitrogen) from human mucosal tissues and cells grown for 16 hrs in serum-free DMEM medium. After reverse transcription, the cDNA was hybridized to Affymetrix Human Genome U133A (mucosal human tissues) or U133 Plus 2.0 (SW48 and NIH3T3 cells) GeneChips per manufacture protocol. MAS-3 software was used for obtaining raw data from the gene chips. LGR5 gene expression > 3 SD above normal was considered as significantly gained. Statistical analyses were done with Microsoft Excel, GraphPad Prism 5 and Gene Set Enrichment Analysis 2.0 software (Broad Institute). All statistical tests were two-sided.

### *KRAS* mutation

Genomic DNA was extracted from human colon cancer tissues with a proteinase K/lithium chloride/ethanol protocol (Qiagen). *KRAS* mutation status was assessed by a PCR/ligase detection reaction (LDR) technique as previously described [54]. Primers and Taq DNA polymerase were used to amplify *KRAS* exon 1. Wild-type primers for *KRAS* codon 30 and mutation-specific primers for codons 12 and 13 were used in the LDR. The LDR products were resolved on a 12.5% polyacrylamide gel in an ABI PRISM 377 DNA Sequencer.

## SUPPLEMENTARY MATERIAL FIGURE AND TABLES




