# Supplementary material for: Oncogenic KRAS activates an embryonic stem cell-like program in human colon cancer initiation
Source: Oncotarget. 2016 Jan 4;7(3):2159–74. doi: 10.18632/oncotarget.6818 (PMC4823026; doi:10.18632/oncotarget.6818)
Supplement: Supplementary file 1 [file oncotarget-07-2159-s001.pdf]

Oncogenic KRAS activates an embryonic stem cell-like program in human colon cancer initiation

Supplementary Material

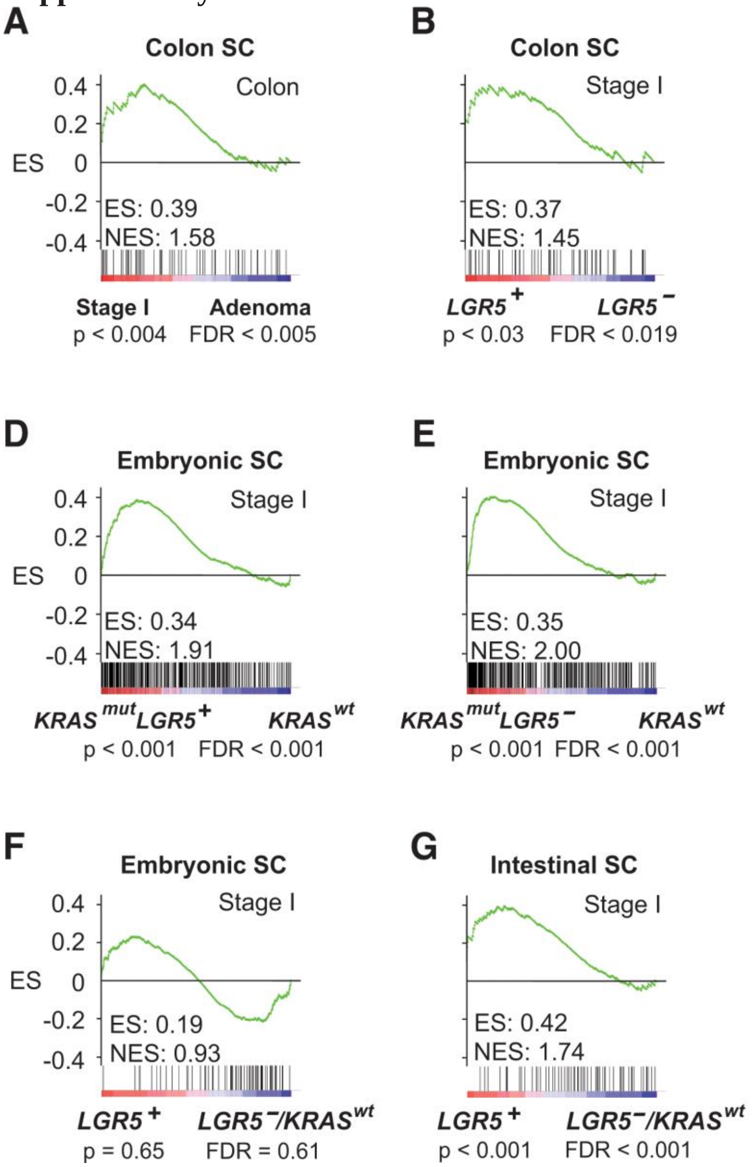

Supplemental Figure 1. A, GSEA on stage I colon carcinomas versus adenomas using a human colon SC signature [55]. **B-G,** GSEA on human stage I colon carcinomas stratified to *LGR5*, *KRAS* and combined *LGR5*/*KRAS* status as indicated using the colon SC (**B, C**), embryonic SC-like (**D-F**) and intestinal SC (**G**) signatures. ES, enrichment score; NES, normalized enrichment score; FDR, false discovery rate. **H,** Representative dark field images of *in situ* hybridization with ^{33}P-probe specific for human *LGR5* mRNA and their corresponding H&E stainings showing specific *LGR5*$^{+}$ intestinal SC localization to the hyperplastic crypt bases of human colon adenoma and normal small intestine, respectively.

Supplemental Table 1. List of the genes in the embryonic SC-like, embryonic SC-like minus proliferation and *MYC*-related target genes, intestinal SC and EMT gene signatures used in GSEA.

Supplemental Table 2. List of the top 80 $KRAS^{mut}$ leading-edge genes enriched in the embryonic SC-like gene signature from GSEA of lenti-$KRAS^{mut}$ versus lenti-pCCL SW48 cells.

Supplemental Table 3. The median and mean *LGR5* mRNA expression levels in normal and malignant human tissues.
